# Alcohol use and associated factors among adolescent boys and young men in Kampala, Uganda

**DOI:** 10.1186/s13011-021-00385-8

**Published:** 2021-06-09

**Authors:** Steven Ndugwa Kabwama, Joseph KB Matovu, John M Ssenkusu, Tonny Ssekamatte, Rhoda K. Wanyenze

**Affiliations:** 1 Department of Disease Control and Environmental Health, Makerere University School of Public Health, Kampala, Uganda; 2 Department of Community and Public Health, Busitema University Faculty of Health Sciences, Mbale, Uganda; 3 Department of Epidemiology and Biostatistics, Makerere University School of Public Health, Kampala, Uganda

**Keywords:** Alcohol, adolescents, young men, Uganda

## Abstract

**Background:**

Alcohol use leads to about 3 million deaths globally. The alcohol industry employs marketing strategies to establish their brands in the lives of young people at a time when addictive behaviors are initiated and reinforced. We conducted a survey among adolescent boys and young men (ABYM) to estimate the prevalence of alcohol use and associated factors using the Health Belief Model as the guiding framework.

**Methods:**

The study was conducted among ABYM in- or out-of-school aged 10–24 years in Kampala, Uganda. We used questions adopted from the Global School-based Student Health Survey and the WHO STEPwise approach to Surveillance questionnaire to collect data. The outcome of interest was alcohol use within 30 days before the interview. We also asked about characteristics such as alcohol use by siblings, parents/ guardians, school status among others. We used odds ratios obtained via a logistic regression model as the measure of association.

**Results:**

A total of 2500 ABYM participated, of which 262 (10.5 %, 95 %CI 9.3–11.7) had consumed alcohol within 30 days before the interview. Out-of-school ABYM had higher odds of consuming alcohol compared with their in-school counterparts AOR 1.55 (95 %CI 1.09–2.20). Compared with ABYM whose parents/ guardians did not drink alcohol, ABYM whose both parents consumed alcohol had higher odds of consuming alcohol AOR 2.24 (95 %CI 1.38–3.64) as were those with only a mother or female guardian who consumed alcohol AOR 1.95 (95 %CI 1.11–3.41). ABYM with siblings that drink alcohol had higher odds of consuming alcohol AOR 2.25 (95 %CI 1.80–3.52). ABYM who possessed items with an alcohol brand logo had higher odds of consuming alcohol AOR 2.00 (95 %CI 1.33–3.01).

**Conclusions:**

There are significant levels of alcohol consumption among ABYM which calls for evidence-based measures targeting this age group to reduce consumption and recognizing the role of the family, school and community in prevention and promotion of use. There is need to regulate alcohol marketing and ensuring availability of alcohol dependence treatment services that build confidence among youth.

## Background

According to the World Health Organization Global Status Report on Alcohol and Health, harmful alcohol use led to about 3 million deaths globally in 2016 [1]. The contribution of alcohol consumption to global mortality was estimated to be 5.3 % which is higher than mortality due to tuberculosis (2.3 %), HIV/AIDS (1.8 %), diabetes (2.8 %) and road injuries (2.5 %). Despite its significant contribution to the global burden of disease, alcohol consumption is anticipated to increase over the next decade. An analysis of current trends of alcohol consumption globally projected that alcohol abstention would reduce from 43 % to 2017 to 40 % in 2030 while the proportion of current drinkers would increase from 47 % to 2017 to 50 % in 2030 [2]. This increase in the number of alcohol consumers has been attributed to the tactical and deliberate marketing strategies employed by the alcohol industry to establish and entrench their brands in the lives of people especially the youth [3]. The second decade of life is a critical time during the life course of development because it is the window during which behavior that continues into adulthood is initiated and reinforced. Adolescents and young people are thus particularly vulnerable to the social and biological immediate and long term effects of alcohol consumption. A review of the effects of alcohol use on the brain and behaviour among young people noted that alcohol use was associated with difficulties in verbal learning, visual-spatial processing as well as deficits in the development of the central nervous system [4]. The result of these effects include a reduction in cognitive flexibility, behavioural inefficiencies, increased anxiety, disinhibition and risk taking. In addition, alcohol use is associated with other direct and indirect long term effects such as liver disease [5] and obesity [6] particularly among men [7]. There has been a steady increase in the prevalence of obesity in Uganda from 8 % to 1995 [8], 18.8 % in 2011 [9] and 24 % in 2016 [10].

In 2014, a national survey in Uganda established that 26.8 % of adults used alcohol in the past month and 9.8 % had an alcohol use disorder [11]. Although the study did not report about the age of initiation of use, previous research has demonstrated that addictive behaviour such as alcohol and tobacco use are initiated between the ages 10–18 [12–14]. In addition to the vulnerability due to age, adolescent boys and young men are more likely to engage in high-risk and violent behaviours than their female counterparts [15, 16]. For instance, compared with females, males are more likely to engage in binge drinking and to drive a vehicle while intoxicated [17]. Males are also more likely to engage in risky sexual behavior after drinking alcohol [18]. Furthermore, about one third of the world’s urban population live in neighborhoods characterized by lack of decent housing, preventable diseases, illiteracy, violence and crime [19]. However, while evidence suggests that the growing challenges of urban residence are greater among men than women [20], few studies have included men in general and adolescent boys and young men in particular.

We conducted a survey to assess the risk-taking behaviours of adolescent boys and young men in Kampala, Uganda. Specifically, we aimed to estimate the prevalence of alcohol use and associated factors using the Health Belief Model [21] as the guiding framework. Findings will inform the development of interventions and strategies to reduce alcohol use in this specific age group.

## Methods

### Study site

The study was conducted among adolescent boys and young men within the five divisions of Kampala, the Capital City of Uganda with an estimated population of 1,650,800 [22]. The five divisions of Kampala City include Kawempe, Rubaga, Makindye, Nakawa and Central Division.

### Study design

This was a cross-sectional study that employed quantitative data collection methods.

### Study population

The study population included adolescent boys and young men aged 10–24 years. All adolescent boys and young men in any of the 5 divisions of Kampala aged 10–24 years and who were either in- or out-of-school were included in the study except those who failed to provide consent or did not understand English or Luganda. In-school adolescent boys and young men were those in the official school-age group and enrolled in any form of education while out-of-school ABYM were those in the official school-age group and not enrolled in any education for a period of at least one year preceding the date of the interview including drop outs and those who completed secondary education but lacked the resources to pursue post-secondary education, or who were unemployed or under employed [23].

### Sample size and sampling considerations

The detailed sample size computation and sampling procedures have been described in a previous publication [24]. Briefly, we considered a type-1 error of 5%, p = 0.14 (the proportion of adolescent boys that have ever used drugs in Kampala, Uganda) [25]; design effect of 2.0; a margin of error of 0.05, 5 divisions and a non-response of 0.10, to obtain minimum sample of 2,060 ABYM. We however adjusted this upwards to 2500 ABYM and used probability proportionate to size to allocate the sample size across the 5 divisions of Kampala.

### Sampling strategy

Participants were enrolled into the study at household level. Household interviews were conducted in selected villages in all the five divisions of Kampala. We used a multi-stage sampling technique at the parish, village and household level to select the study participants.

ABYM who were studying in a school that was not located in Kampala were excluded from the study.

Since there was no sampling frame for out of school ABYM, we used the Lot Quality Assurance Sampling (LQAS) methodology [26] to sample out of school ABYM from areas of location and/or types of occupation (such as garages, boda-boda [motorcycle taxi] stages, mobile traders, quarries, construction sites). Locations and/or types of occupations were considered as sampling lots and we enrolled a minimum of 19 respondents from each lot within a ward until the required number of out-of-school ABYM in each division was attained [24].

### Data collection

Data were collected between July and August 2020. We adopted the Global School-based Student Health Survey (GSHS) [27], a WHO validated self-administered questionnaire that collects information on health behaviors of school pupils including alcohol use. The GSHS is designed to capture data on students between 13 and 17 years. The questionnaire was expanded to collect data from out-of-school ABYM, age groups 10–24 years, and modified to an interview-administered questionnaire. Age was assessed as self-reported number of complete years. Other questions on alcohol use were adapted from the WHO STEPwise approach to Surveillance questionnaire [28].

Alcohol use was assessed by asking participants whether they had ever consumed any alcohol, whether they consumed alcohol within the previous 12 months or previous 30 days.

According to the Health Belief Model, engagement in behaviour is predicted by *risk perception*, *perceived benefit of engagement in the behaviour*, *perceived barriers*, *cues to action* [21] and *self-efficacy* [29]. Perception of risk as a predictor to engagement in behaviour is done considering a specific outcome (hazard). In this study, we considered obesity as the hazard because of (a) the high level of alcohol use in Uganda [11], (b) the documented association between alcohol consumption and weight gain [6] particularly among men [7], and (c) the steady increase in the prevalence of obesity in Uganda from 8 % to 1995 [8], 18.8 % in 2011[9] and 24 % in 2016 [10]. We assessed *risk perception* for obesity by adapting a multi-dimensional tool that incorporates likelihood, vulnerability and salience of risk judgments to quantify perceived risk [30]. We assessed *perceived barriers* by asking participants the extent to which they thought it would be difficult to limit alcohol consumption to 2 standard drinks or less per day. A standard drink of alcohol contains 10 g of pure alcohol [31] and pictures of commonly drank alcoholic beverages equivalent to a standard drink were used during the training of the research assistants so that they could explain to interviewees to understand the quantity of a standard alcoholic drink. Category 1 adult alcohol users are those who consume less than 39.9 g of pure alcohol or 4 standard drinks in a day [31]. For this age group however, we considered the ability to limit consumption to less than 2 standard drinks a day. *Self-efficacy* was assessed by asking participants to use a scale to rate how certain they were that they would limit their alcohol consumption to 2 standard drinks or less per day. *Perceived benefits* were assessed by asking participants whether they thought it would be beneficial to their health to limit alcohol consumption to 2 standard drinks or less and if so the extent to which this would be beneficial. *Cues to action* were those already contained in the Global School-based Health Survey tool and included alcohol advertisement and promotion as well as alcohol use behaviour by parents and siblings.

### Statistical analysis

We presented the socio-demographic characteristics using proportions. The items on the risk-perception tool were each scored with 1 for “yes” and 0 for “no” and higher scores indicated higher risk perception. The Pearson’s Chi-square statistic was used to test the difference in risk factors of alcohol use between groups by school status and age. The null hypothesis was that for each of the risk factors, the proportion of ABYM by school status and age was the same across the sub-categories. This hypothesis was rejected at a p-value ≤ 0.05. We also estimated the effect sizes of the associations between the categorical variables using Phi [*φ*] for binary categorical variables and the Cramer’s V [*φ*_*c*_] for larger group comparisons.

The main outcome of interest was alcohol use within the 30 days that preceded the interview. To determine the association between alcohol use within the previous 30 days and the predictor variables, we used odds ratios obtained via a logistic regression model as the measure of association. The association analysis was conducted in two steps. In the first step, we fitted bivariate logistic regression models for each predictor variable and alcohol use to obtain crude odds ratios. In the second step, all predictor variables with a p-value < 0.1 at bivariate analysis were included in an adjusted model to obtain adjusted odds ratios (AOR). The predictor variables included in the model were informed by the health belief model i.e. perception of risk for obesity, perceived benefits of reducing alcohol use, perceived barriers, cues to action such alcohol use behaviour by siblings and parents and self-efficacy. Other variables outside of this framework but included in the model were school-going status and age. All analyses were done in STATA version 13 (StataCorp, College Station, Texas USA).

## Results

### Characteristics of participants in the survey

A total of 2500 Adolescent Boys and Young Men participated of whom 1869 (74.8 %) were in school at the time of the survey (Table 1). Among those in school, 1303 (69.3 %) resided at their homes during school, while the rest reside in hostels, halls of residence or other housing at school. Almost 6 in 10 participants 1458 (58.3 %) were Baganda ethnic group.

**Table 1:** Socio-demographic characteristics of Adolescent Boys and Young Men who participated in the survey

Variable	-n-	%
Total	2500	100
School going status		
In-school	1869	74.8
Out-of-school	631	25.2
Class at time of interview (among those in-school)		
<P5	158	8.5
P6-P7	262	14.0
S1-S2	346	18.5
S3-S4	481	25.7
S5-S6	397	21.2
Tertiary/ University	225	12.0
Residence during school		
School	437	23.4
Home	1303	69.7
Hostel/ Hall of residence	129	6.9
Division		
Central	257	10.3
Makindye	405	16.2
Nakawa	720	28.8
Kawempe	617	24.7
Rubaga	501	20.0
Age-group		
10–14	483	19.3
15–19	1182	47.3
20–24	835	33.4
Tribe		
Baganda	1458	58.4
Others	1042	41.6

### Alcohol use among ABYM in the survey

Of the 2500 ABYM that participated in the survey, 771 (30.8 %) had ever consumed alcohol while 262 (10.5 %, 95 %CI 9.3–11.7) had consumed alcohol within the 30 days preceding the date of the interview (Table 2). Almost half (1244, 49.8 %) had seen “A lot” of alcohol advertisements on TV in the 30 days preceding the date of the interview while 298 (10.8 %) owned an item with an alcohol brand logo on it.

**Table 2:** Alcohol Use among Adolescent Boys and Young Men by School-going Status and Age Category

	Total	School Status		Chi (-p-) [*φ*] or [*φ*_*c*_]	Age Category			Chi (-p-) [*φ*] or [*φ*_*c*_]
**Variable**		**In** **n = 1869 (%)**	**Out** **n = 631 (%)**		**10–14** **n = 483 (%)**	**15–19** **n = 1182 (%)**	**20–24** **n = 835 (%)**	
Ever consumed any alcohol								
No	1729 (69.2)	1426 (76.3)	303 (48.0)	176.9 (< 0.05) [*φ* = 0.27]	458 (94.8)	865 (73.2)	406 (48.6)	323.2 (< 0.05) [*φ*_*c*_ = 0.36]
Yes	771 (30.8)	443 (23.7)	328 (52.0)		25 (5.2)	317 (26.8)	429 (51.4)	
Consumed alcohol within past 12 months								
No	2040 (81.6)	1624 (86.9)	416 (65.9)	8.2 (0.004) [*φ* = 0.10]	468 (96.9)	1026 (86.8)	546 (65.4)	24.9 (< 0.05) [*φ*_*c*_ = 0.18]
Yes	460 (18.4)	245 (13.1)	215 (34.1)		15 (3.1)	156 (13.2)	289 (34.6)	
Consumed any alcohol in past 30 days								
No	2238 (89.5)	1745 (93.4)	493 (78.1)	116.7 (< 0.05) [*φ* = 0.22]	477 (98.8)	1104 (93.4)	657 (78.7)	167.4 (< 0.05) [*φ*_*c*_ = 0.26]
Yes	262 (10.5)	124 (6.6)	138 (21.9)		6 (1.2)	78 (6.6)	178 (21.3)	
Number of alcohol advertisements seen on TV in past 30 days								
A lot	1244 (49.8)	902 (48.3)	342 (54.2)	7.8 (0.020) [*φ*_*c*_ = 0.06]	215 (44.5)	594 (50.3)	435 (52.1)	11.6 (0.020) [*φ*_*c*_ = 0.05]
None	376 (15.1)	282 (15.1)	94 (14.9)		93 (19.2)	164 (13.9)	119 (14.2)	
A few	880 (35.2)	685 (36.7)	195 (30.9)		175 (36.2)	424 (35.9)	281 (33.7)	
Parents or guardians that drink alcohol								
Neither/ I don’t know	1598 (63.9)	1211 (64.0)	387 (61.3)	3.8 (0.287) [*φ*_*c*_ = 0.04]	339 (70.2)	742 (62.8)	517 (61.9)	13.6 (0.035) [*φ*_*c*_ = 0.05]
Father or male guardian	525 (21.0)	376 (20.1)	149 (23.6)		82 (17.0)	266 (22.5)	177 (21.2)	
Mother or female guardian	173 (6.9)	131 (7.0)	42 (6.7)		28 (5.8)	85 (7.2)	60 (7.2)	
Both	204 (8.2)	151 (8.1)	53 (8.4)		34 (7.0)	89 (7.5)	81 (9.7)	
Any siblings that drink alcohol								
No	1900 (76.4)	1492 (79.8)	417 (66.1)	49.4 (< 0.05) [*φ* = 0.14]	444 (91.9)	936 (79.2)	529 (63.4)	148.3 (< 0.05) [*φ*_*c*_=]
Yes	591 (23.6)	377 (20.2)	214 (33.9)		39 (8.1)	246 (20.8)	306 (36.6)	
Number of friends that drink alcohol								
None	1055 (42.2)	945 (50.6)	110 (17.4)	244.6 (< 0.05) [*φ*_*c*_ = 0.31]	398 (82.4)	527 (44.6)	130 (15.6)	626.8 (< 0.05) [*φ*_*c*_ = 0.24]
Some/ A few	1043 (41.7)	706 (37.8)	337 (53.4)		76 (15.7)	520 (44.0)	447 (53.5)	
Most	346 (13.8)	194 (10.4)	152 (24.1)		8 (1.7)	120 (10.2)	218 (26.1)	
All	56 (2.2)	24 (1.3)	32 (5.1)		1 (0.2)	15 (1.3)	40 (4.8)	
Possess items with alcohol brand logo								
No	2231 (89.2)	1771 (91.6)	520 (82.4)	41.0 (< 0.05) [*φ* = 0.13]	465 (96.3)	1068 (90.4)	698 (83.6)	54.1 (< 0.05) [*φ*_*c*_ = 0.15]
Yes	269 (10.8)	158 (8.4)	111 (17.6)		18 (3.7)	114 (9.6)	137 (16.4)	

Although 1 in 5 in-school ABYM (443, 23.7 %) had ever consumed alcohol, more than half of the out-of-school ABYM had ever consumed alcohol (328, 52 %). The distribution of alcohol use by age category showed that more than half of respondents aged 20–24 years 429 (51.4 %) had ever consumed alcohol. In addition, 317 (26.8 %) of those aged 15–19 had ever consumed alcohol while 25 (5.2 %) of those aged 10–14 had ever consumed alcohol. Among those aged 10–14 years at the time of the survey, 6 (1.2 %) had consumed alcohol within 30 days preceding the date of the interview. The Pearson’s Chi-square test statistic revealed statistically significant differences in alcohol consumption between groups by school status and age across all the categorical variables assessed except consumption of alcohol by parents/ guardians.

### Factors associated with alcohol consumption among adolescent boys and young men in Kampala

Adolescent boys and young men who were out of school had higher odds of consuming alcohol compared with their in-school counterparts AOR 1.55 (95 %CI 1.09–2.20) (Table 3). Compared with ABYM whose parents/ guardians did not drink alcohol, ABYM whose both consumed alcohol had significantly higher odds of consuming alcohol AOR 2.24 95 %CI (1.38–3.64) as were those with only a mother or female guardian who consumed alcohol AOR 1.95 95 %CI (1.11–3.41). ABYM who had siblings that drink alcohol also had significantly higher odds of consuming alcohol AOR 2.25 95 %CI (1.80–3.52).

**Table 3:** Crude and Adjusted Odds Ratios of Alcohol Use among Adolescent Boys and Young Men in Kampala

	Crude OR (95 % CI)		Adjusted OR (95 % CI)	p-value
**Variable**				
School-going status				
In-school	1.0		1.0	
Out-of-school	3.94 (3.03–5.12)	< 0.05	1.55 (1.09–2.20)	**0.014**
Age category				
10–14	1.0		1.0	
15–19	5.62 (2.43–12.98)	< 0.05	0.62 (0.22–1.73)	0.363
20–24	21.54 (9.47-49.00)	< 0.05	1.15 (0.41–3.19)	0.800
Parents or guardians that drink alcohol				
Neither/ Don’t know	1.0		1.0	
Both	6.15 (4.26–8.87)	< 0.05	2.24 (1.38–3.64)	**0.001**
Father or male guardian	2.62 (1.91–3.60)	< 0.05	1.29 (0.86–1.92)	0.212
Mother or female guardian	3.38 (2.18–5.25)	< 0.05	1.95 (1.11–3.41)	**0.020**
Any siblings that drink alcohol				
None/ No siblings/ Don’t know	1.0		1.0	
Yes	7.52 (5.72–9.88)	< 0.05	2.25 (1.80–3.52)	**< 0.05**
Number of alcohol adverts seen on TV in past 30 days				
None	1.0		1.0	
A few	1.09 (0.71–1.67)	0.694	0.82 (0.47–1.43)	0.486
A lot	1.46 (0.98–2.18)	0.063	0.71 (0.42–1.23)	0.223
Number of alcohol adverts seen on billboards in past 30 days				
None	1.0		1.0	
A few	1.46 (1.07–1.99)	0.017	0.95 (0.64–1.42)	0.816
A lot	2.46 (1.76–3.45)	< 0.05	1.09 (0.70–1.70)	0.706
Possess items with alcohol brand logo				
No	1.0		1.0	
Yes	4.23 (3.11–5.74)	< 0.05	2.00 (1.33–3.01)	**0.001**
Personal certainty about limiting alcohol consumption				
Highly certain	1.0		1.0	
Moderately certain	3.01 (2.18–4.16)	< 0.05	2.47 (1.74–3.51)	**< 0.05**
Cannot at all	3.15 (1.70–5.83)	< 0.05	2.32 (1.19–4.51)	**0.013**
Obesity risk score	0.99 (0.97–1.02)	0.635		Omitted

ABYM who possessed items with an alcohol brand logo on it had significantly higher odds of consuming alcohol AOR 2.00 95 %CI (1.33–3.01). Compared with ABYM who were certain that they could limit their alcohol consumption, those who were only moderately certain had higher odds of consuming alcohol AOR 2.47 95 %CI (1.74–3.51) as were those who were not certain at all that they could limit their alcohol consumption AOR 2.32 95 % CI (1.19–4.51).

Compared with participants who had not seen any alcohol adverts on TV in the past 30 days, those who had seen a few AOR 0.82; 95 % CI (0.47–1.43) and those who had seen a lot AOR 0.71; 95 % CI (0.42–1.23) did not have significantly higher odds of having drank alcohol in the past 30 days. Similarly, compared with participants who had not seen any alcohol adverts on billboards in the past 30 days, those who had seen a few AOR 0.95; 95 % CI (0.64–1.42) and those who had seen a lot AOR 1.09; 95 % CI (0.70–1.70) did not have significantly higher odds of having drank alcohol in the past 30 days.

## Discussion

The study aimed to estimate the prevalence of alcohol use and associated factors using the Health Belief Model. The findings from the survey revealed significant levels of alcohol consumption and exposure to alcohol advertising and promotion among the ABYM. More than 1 in 3 had ever consumed alcohol, 1 in 10 had consumed alcohol in the 30 days preceding the interview, almost half (1244, 49.8 %) had seen “A lot” of alcohol advertisements on TV in the 30 days preceding the survey and more than 1 in 10 owned an item with an alcohol brand logo on it. These findings are consistent with those in a survey among 457 male and female out-of-school youth in neighbourhoods of Kampala City in which more than 1 in 3 reported problem drinking and drunkenness and 62.1 % had been exposed to alcohol advertisement and promotion [32]. ABYM who possessed items with an alcohol brand logo on it had significantly higher odds of consuming alcohol. The results of the analysis also showed that although there was a positive albeit non-significant association between having seen alcohol adverts on TV in the past 30 days and alcohol use and a significant association between having seen an alcohol advert on billboards in the past 30 days and alcohol use in the crude analysis, there was no association in the adjusted analysis. In contrast, owning items with alcohol brands was associated with alcohol use, both independently and when controlling for other social-cognitive alcohol risk factors. This might be attributed to a selection effect where youth already interested or already using alcohol were the ones that had the items branded with alcohol company logos. Alcohol advertisement, promotion and sponsorship have a direct effect on early initiation of use, frequency and quantity of alcohol consumption and may explain the heavy burden of alcohol use among adults in Uganda [11]. Indeed, a cohort study on the impact of alcohol marketing on youth drinking behaviour demonstrated that alcohol marketing at baseline was predictive of both the initiation of alcohol consumption and frequency of drinking [33]. In this age-group however, alcohol adverts maybe a distal risk factor particularly compared to risk factors like alcohol use by other members of the family and social-cognitive factors like low self-efficacy for limiting alcohol and high barriers to limiting alcohol. There is a need for the establishment of a legal framework that will protect the youth from the influence of the alcohol industry through strategies such as prohibition of sale to minors, prohibition of alcohol in education institutions, limiting density of outlets, limiting alcohol promotion and sponsorship among others. An assessment by WHO established that banning alcohol advertising to reduce alcohol consumption is cost effective and can have incremental benefits when coupled with taxation [34].

Adolescent boys and young men who were out of school had higher odds of consuming alcohol compared with their in-school counterparts. Swahn and colleagues have reported similar findings of high alcohol consumption among youth who are out of school in Kampala City [32, 35]. A similar assessment among 132,600 youth noted higher levels of alcohol and illicit drug use among those that dropped out of school compared with those who were in school [36]. Although the design of the current study precludes conclusions on the direction of causality between schooling status and alcohol use, a previous study showed that youth who are out of school are at a significantly higher risk of adverse health outcomes such alcohol use because of the influence of the disadvantaged living environment [37] that facilitates risky behaviour. Policy makers, youth leaders, parents, teachers, school administrators need to be informed about the problem of alcohol use among ABYM in general and those who drop out of school in particular to develop innovative ways of creating alcohol free neighbourhoods while keeping boys in school.

The study has also shown that compared with ABYM whose parents/ guardians and siblings did not drink alcohol, ABYM who had parents/ guardians or siblings that consume alcohol had significantly higher odds of consuming alcohol. According to the Health Belief Model, engagement in behaviour is predicted by risk perception, perceived benefit of engagement in the behaviour, self-efficacy, perceived barriers and cues to action [21]. Cues to action refer to the internal and external enablers that facilitate engagement in certain behaviour. In this case, alcohol use by siblings and parents/ guardians is a cue that inadvertently certifies and inculcates positive attitudes towards the behaviour to the ABYM at an age at which they are vulnerable to being influenced by what they experience in their environment. Familial alcohol problems have been previously documented to be significant predictors of alcohol use among youth [38]. Strategies for control and prevention of alcohol use among ABYM should adopt a whole of society approach by recognizing the role of the family, school and the community at large in the prevention of alcohol use in this age group.

The Health Belief Model also posits that self-efficacy or an individual’s confidence in their ability to engage in a certain behaviour is an important predictor for engagement in that behaviour [29]. Compared with ABYM who were certain that they could limit their alcohol consumption, those who were less certain had higher odds of consuming alcohol. Deficiencies in confidence to limit alcohol use could also be explained by the effects of the marketing strategies by the alcohol industry where almost half of our respondents (1244, 49.8 %) had seen “A lot” of alcohol advertisements on TV in the 30 days preceding the survey and more than 1 in 10 owned an item with an alcohol brand logo on it. A prospective study conducted among men and women on treatment for alcohol dependence found that higher self-efficacy scores were associated with better outcomes such as lower likelihood of consuming alcohol [39]. There is a need to enact legislation that protects young people from exposure to deceptive marketing strategies that glorify alcohol use. This also calls for the availability of alcohol dependence treatment services that incorporate aspects of building confidence among the youth such as addressing mental health challenges like depression, and enhancing social support [40].

## Limitations

The major limitation of the current study was the design where both predictor and outcome variables were assessed simultaneously. The other limitation was that data about the dependent variable on alcohol use were obtained using self-reports which introduces bias. In addition, the study considered a long term hazard of alcohol use (obesity) and yet there are other immediate and direct effects of alcohol use such as engagement in risky sexual behaviour [18] and alcoholic liver disease [5]. In addition, the findings from the current study are not generalizable to girls or non-binary youth. However, we collected data from a significant sample size and used validated tools to obtain information. Both these factors lend credence to the interpretation of the findings and conclusions reached.

## Conclusions

This survey has revealed high levels of alcohol consumption among adolescent boys and young men during a stage of life when addictive behaviors are initiated and reinforced. Public health interventions targeted at specific behaviors such as alcohol use during adolescence are likely to have benefits that will not only continue into adulthood but also reduce the prevalence of the behavior among adolescents in the long term [41]. Alcohol use was significantly associated with cues to action such as alcohol use by friends, siblings or parents/ guardians. Strategies for control and prevention of alcohol use among ABYM should adopt a whole of society approach by recognizing the role of the family, school and the community at large in the prevention of alcohol use in this age group. Lack of self-efficacy or an individual’s confidence in their ability to limit alcohol use was also an important predictor for use. There is a need to regulate alcohol marketing including the content and ensuring the availability of alcohol dependence treatment services that incorporate aspects of building confidence among the youth.

## Data Availability

The dataset used for analysis can be availed upon reasonable request by writing an email to the corresponding author.

## Abbreviations

ABYM: Adolescent Boys and Young Men
AOR: Adjusted Odds Ratios
GSHS: Global School Health Survey
STEPS: STEpwise Approach to Surveillance
WHO: World Health Organization

## References

[1] World Health Organization (WHO). Global status report on alcohol and health 2018. World Health Organization; 2019.

[2] Manthey J, Shield KD, Rylett M, Hasan OS, Probst C, Rehm J (2019). Global alcohol exposure between 1990 and 2017 and forecasts until 2030: a modelling study. Lancet.

[3] Jernigan DH (2010). The extent of global alcohol marketing and its impact on youth. Contemp Drug Probl.

[4] Spear LP (2018). Effects of adolescent alcohol consumption on the brain and behaviour. Nat Rev Neurosci.

[5] Diehl AM (2002). Liver disease in alcohol abusers: clinical perspective. Alcohol.

[6] Traversy G, Chaput J-P (2015). Alcohol consumption and obesity: an update. Curr Obes Rep.

[7] Shelton NJ, Knott CS (2014). Association between alcohol calorie intake and overweight and obesity in English adults. Amer J Pub Health.

[8] Ministry of Finance. Planning and Economic Development, Uganda, Macro International. Uganda Demographic and Health Survey 1995. Maryland: Statistics Department/Uganda and Macro International;: Calverton; 1996.

[9] Uganda Bureau of Statistics (UBOS) (2012). The Uganda Demographic and Health Survey 2011. Kampala Uganda: UBOS and Calverton.

[10] Uganda Bureau of Statistics (UBOS). Uganda Demographic Health Survey 2016. Kampala: UBOS and Calverton, Maryland: ICF International Inc;; 2017.

[11] Kabwama SN, Ndyanabangi S, Mutungi G, Wesonga R, Bahendeka SK, Guwatudde D. Alcohol use among adults in Uganda: findings from the countrywide non-communicable diseases risk factor cross-sectional survey. Glob Health Action. 2016;9. 10.3402/gha.v9.31302 PMC4974493 27491961

[12] Faden VB (2006). Trends in initiation of alcohol use in the United States 1975 to 2003. Alcohol Clin Exp Res.

[13] Everett SA, Warren CW, Sharp D, Kann L, Husten CG, Crossett LS (1999). Initiation of cigarette smoking and subsequent smoking behavior among US high school students. Prev Med.

[14] Everett SA, Husten CG, Kann L, Warren CW, Sharp D, Crossett L (1999). Smoking initiation and smoking patterns among US college students. J Amer Coll Health.

[15] Mealey L. Sex differences: Developmental and evolutionary strategies. Academic Press; 2000.

[16] Gupta N, Simonsen M. Non-cognitive child outcomes and universal high quality child care. IZA Discussion Papers 3188. Institute for the Study of Labor (IZA). 2007.

[17] Elster AB, Marcell AV (2003). Health care of adolescent males: overview, rationale, and recommendations. Adol Med Clin.

[18] Graves KL (1995). Risky sexual behavior and alcohol use among young adults: Results from a national survey. Amer J Health Prom.

[19] UN Habitat UNHSPU. The State of the World’s Cities 2006/7, The Millenium Development Goals and Urban Sustainability. London Earthscan; 2006.

[20] Springer AE, Selwyn BJ, Kelder SH (2006). A descriptive study of youth risk behavior in urban and rural secondary school students in El Salvador. BMC Int Health Hum Rights.

[21] Becker MH (1974). The health belief model and personal health behavior. Health Educ Mon.

[22] Uganda Bureau of Statistics (UBOS). National Population and Housing Census 2014 Revised Edition. Kampala; 2014.

[23] Kerka S, Connection OSUL (2006). Out-of-school youth.

[24] Matovu JK, Kabwama SN, Ssekamatte T, Ssenkusu J, Wanyenze RK. COVID-19 Awareness, Adoption of COVID-19 Preventive Measures, and Effects of COVID-19 Lockdown Among Adolescent Boys and Young Men in Kampala, Uganda. J Comm Health. 2021:1–12. 10.1007/s10900-021-00961-w PMC7820821 33481156

[25] Swahn MH, Buchongo P, Kasirye R (2018). Risky behaviors of youth living in the slums of Kampala: a closer examination of youth participating in vocational training programs. Vul Child Youth Stud.

[26] Lanata CF, Black RE. Lot quality assurance sampling techniques in health surveys in developing countries: advantages and current constraints. World health statistics quarterly 1991; 44 (3): 133–139. 1991. 1949880

[27] World Health Organization. Global school-based student health survey (GSHS). 2013.

[28] Bonita R, Winkelmann R, Douglas KA, de Courten M (2003). The WHO Stepwise approach to surveillance (STEPS) of non-communicable disease risk factors.

[29] Bandura A (1978). Self-efficacy: Toward a unifying theory of behavioral change. Adv Behav Res Ther.

[30] Napper LE, Fisher DG, Reynolds GL. Development of the perceived risk of HIV scale. AIDS Behav. 2012;16(4):1075–83. Barros AJ, Hirakata VN. 10.1007/s10461-011-0003-2 PMC3338003 21785873

[31] Stockwell T, Chikritzhs T, Holder H, Single E, Elena M, Jernigan D (2000). International guide for monitoring alcohol consumption and related harm.

[32] Swahn MH, Palmier JB, Kasirye R. Alcohol exposures, alcohol marketing, and their associations with problem drinking and drunkenness among youth living in the slums of Kampala, Uganda. ISRN Public Health. 2013;2013.

[33] Gordon R, MacKintosh AM, Moodie C (2010). The impact of alcohol marketing on youth drinking behaviour: a two-stage cohort study. Alcohol Alcoholism.

[34] Pan American Health Organization (2017). Technical note: Background on alcohol marketing regulation and monitoring for the protection of public health.

[35] Swahn MH, Culbreth R, Tumwesigye NM, Topalli V, Wright E, Kasirye R (2018). Problem drinking, alcohol-related violence, and homelessness among youth living in the slums of Kampala, Uganda. Int J Environ Res Pub Health.

[36] Tice P, Lipari RN, Van Horn SL. Substance use among 12th grade aged youths, by dropout status. The CBHSQ report. 2017. 29035491

[37] Swahn MH, Culbreth RE, Staton CA, Self-Brown SR, Kasirye R (2017). Alcohol-related physical abuse of children in the slums of Kampala, Uganda. Int J Environ Res Pub Health.

[38] Olsson G, Brolin Låftman S, Modin B (2019). Problematic familial alcohol use and adolescents’ heavy drinking: can conditions in school compensate for the increased risk of heavy drinking among adolescents from families with problematic alcohol use?. Int J Adol Youth.

[39] Greenfield SF, Hufford MR, Vagge LM, Muenz LR, Costello ME, Weiss RD (2000). The relationship of self-efficacy expectancies to relapse among alcohol dependent men and women: a prospective study. J Stud Alcohol.

[40] McKellar J, Ilgen M, Moos BS, Moos R (2008). Predictors of changes in alcohol-related self-efficacy over 16 years. J Subst Abuse Treat.

[41] Ahmad S (2005). Closing the youth access gap: the projected health benefits and cost savings of a national policy to raise the legal smoking age to 21 in the United States. Health Pol.

